# Toxic Factors of Lead and Cadmium Fit in the Ecological Risk Assessment for Microorganisms

**DOI:** 10.3389/fmicb.2022.927947

**Published:** 2022-06-24

**Authors:** Dale Li, Jianwen Chen, Hong Zhang, Xiujuan Zhang, Junjian Li

**Affiliations:** ^1^Institute of Loess Plateau, Shanxi University, Taiyuan, China; ^2^School of Environment and Resources, Shanxi University, Taiyuan, China

**Keywords:** ecological risk assessment, lead, cadmium, toxic factor, microbial diversity

## Introduction

Soil health plays a vital role to sustain plants, animals, and humans (Lehmann et al., 2020). Microbial indicators are superior to physical or chemical indicators in the evaluation of soil health (Fierer et al., 2021), as microorganisms have a short reproductive cycle and are sensitive to environmental changes (Tang et al., 2019; Gorain and Paul, 2021). Researchers have begun to use the abundance and diversity of microorganisms as new ecological evaluation indicators (Li et al., 2020; Schlatter et al., 2022).

The latest nationwide survey of soil contamination in China indicates that 16% of soil sites were polluted primarily with heavy metals (Zhao et al., 2015). Heavy metals are one of the greatest threats to soil health (Yang et al., 2021). The potential ecological risk index (*RI*) is one of the most widely used methods in soil heavy metal contamination assessment (Nag et al., 2022; Wei et al., 2022). However, microbial communities, which show high sensitivity to environmental disturbances, are generally ignored in *RI*. It remains largely unknown whether the applicability of the conventional toxicity factor (TF) for assessing the risk of heavy metals to soil microorganisms. Previous research has shown that the TF of Cu was underestimated when it is applied to assessing the risk to soil bacteria in combined Cu and Cd contamination (Chen et al., 2020). Nevertheless, whether the TF values for other heavy metals need to be adjusted is still controversial. In addition, the abundance and diversity of bacteria and fungi exhibit distinct responses to heavy metal stress (Xiao et al., 2021), therefore, considering only the variation in bacterial parameters limits our understanding of the applicability of TF values.

In this study, we selected Pb with the same TF as Cu to construct a microcosm experiment and designed different Pb (TF = 5) and Cd (TF = 30) concentrations under different ecological risk levels. The purposes of this work were: (1) to investigate the influence of Pb and Cd on microbial abundance and diversity (2) to evaluate whether the TF of Pb and Cd fit in the ecological risk assessment for microorganisms.

## Materials and Methods

We took sample soils for the microcosm construction from a pine forest (*Pinus tabuliformis* Carr.) in the Tianlong Mountain Nature Reserve (37°42'N, 112°27'E), Shanxi Province, China. The determination method of soil physicochemical and heavy metal content (As, Pb, Zn, Cr, Cd, Cu, and Ni) were described in the previous paper (Chen et al., 2020), and soil properties were in Supplementary Table S1. The *RI* was calculated through the following equation (Hakanson, 1980):


$$\begin{array}{l} RI=\sum_{i}^{n}E_{r}^{i}=\sum_{i}^{n}\left(T_{r}^{i}\times \frac{c_{D}^{i}}{c_{R}^{i}}\right) \end{array}$$

where $C_{D}^{i}$ and $C_{R}^{i}\;$are the measured concentrations of heavy metal *i* in the sample and its background reference value (mg kg^−1^), respectively. $T_{r}^{i}$ represents the TF for heavy metal *i*. $E_{r}^{i}\;$denotes the single potential ecological risk factor for heavy metal *i*.

Soil microcosms were constructed according to the risk assessment criteria in Supplementary Table S2. We added fresh soil (equivalent to 40 g dry soil) into 150 ml vials, respectively. The three *RI* levels, low (L, *RI* = 100), moderate (M, *RI* = 200), and high (H, *RI* = 400), were achieved by adding different amount of Pb (CH_3_COO)_2_·3H_2_O and CdCl_2_·2.5H_2_O. There were 5,6 and 6 treatments for three *RI* levels, respectively (Supplementary Table S3). We set original soil as control (CK). Each treatment was with five replicates. All microcosms were incubated at 25°C in the dark. Soil were maintained at 60% water-filled pore space by regularly weighing the vials and replenishing water. Aerobic conditions were maintained by opening microcosm regularly for fresh air exchange. The samples were collected on 45 days for the determination of molecular analysis.

16S rRNA (primer set 338F/518R) and ITS (primer set ITS1F-ITS2R) genes were quantified through qPCR to assess the abundance of bacteria and fungi, respectively. 16S rRNA (primer set 515F/806R) and ITS (primer set ITS1F-ITS2R) genes were determined by high-throughput sequencing to analysis bacterial and fungal communities. High-throughput sequencing and qPCR were performed by Shanghai Majorbio Bio-pharm Technology Co., Ltd, China. Soil microbial diversity was assessed using the Simpson Diversity Index (*D*). The formulas are shown in Supplementary Table S4.

We applied the Biolog Ecoplate and FFplate (Biolog Inc., USA) to analysis the functional diversity of the bacterial and fungal communities, respectively. The measurements were made as previously described (Xie et al., 2021). The absorbance measured at 168 h was used to evaluate the functional diversity of microorganisms, as this showed the optimum range of absorbance (Supplementary Figure S1). The metabolic characteristics of the microbial communities were indicated by the average well color development (AWCD). The functional diversity (utilization of carbon sources) of soil microorganism is assessed using the McIntosh diversity Index (*U*). All formulas are shown in Supplementary Table S4.

The multidiversity index was calculated by averaging the Simpson indices (normalized to between 0 and 1) for bacteria and fungi. The multifunctional index was calculated by averaging the McIntosh indices (normalized to between 0 and 1) for bacteria and fungi. These multifunctionality and multidiversity indices were used widely in the current literature on biodiversity function (Soliveres et al., 2016; Delgado-Baquerizo et al., 2020). The multifunctionality and multidiversity indices are collectively referred to as the Multidiversity index.

Analyses of variance (ANOVA) were performed at the identical and different *RI* levels to test bacterial/fungal abundance, McIntosh diversity (*U*), AWCD, Simpson diversity (*D*) and Multidiversity indices. We used the *ggpubr* package to perform an ANOVA test in R. Correlations between *RI* and microbial parameters were determined using Pearson linear correlation analysis. Pearson correlation analysis were done with SPSS Statistics 22.0. R software (version 3.6.2) was performed to visualize all of the figures.

## Results and Discussion

**Figure 1:**
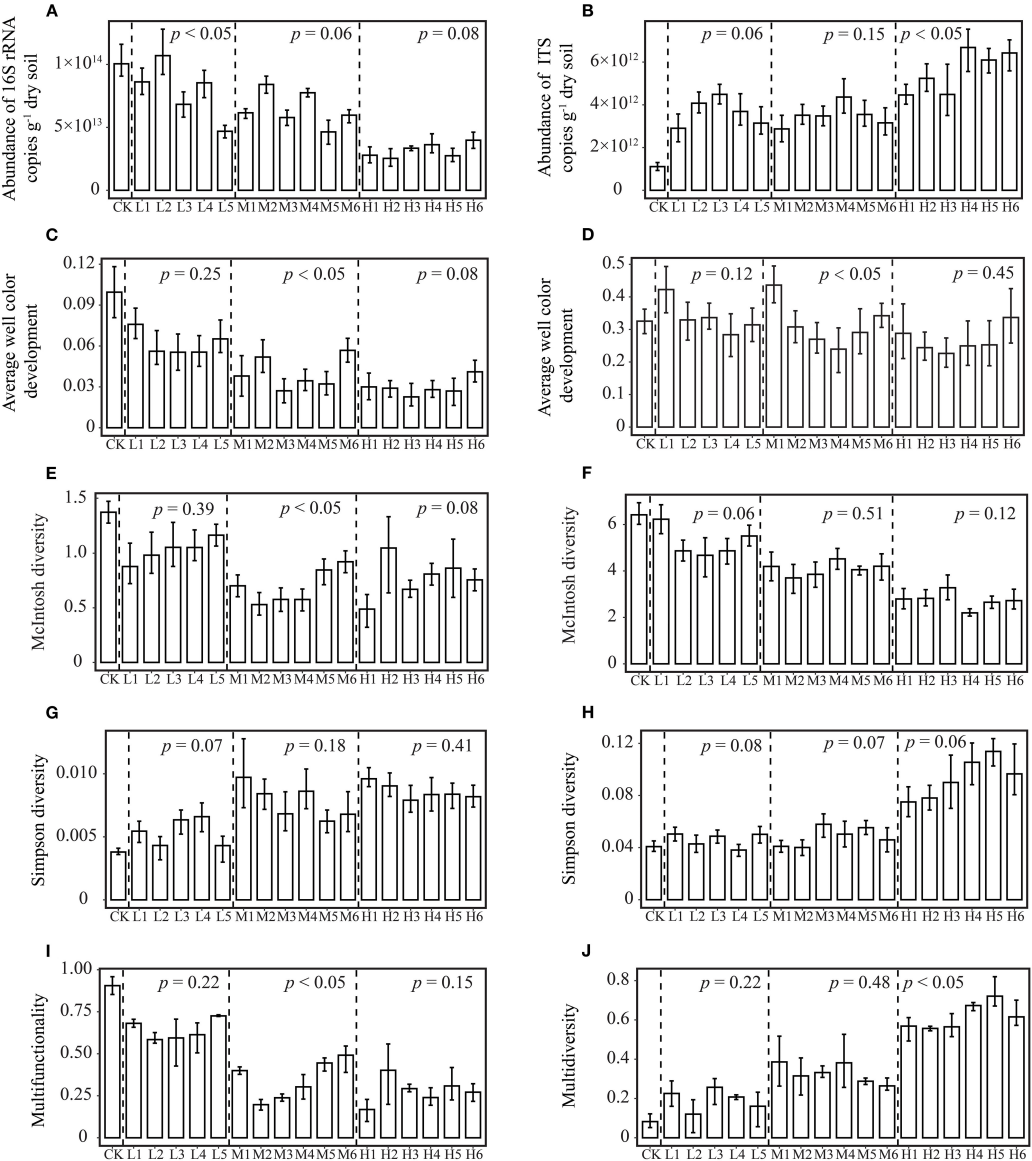
Differences in microbial parameters among different treatments at the same *RI* level. 16S rRNA **(A)** and ITS genes abundance **(B)**; Average well color development in Biolog Ecoplate **(C)** and FFplate **(D)**; McIntosh diversity of bacteria **(E)** and fungi **(F)**; Simpson diversity of bacteria **(G)** and fungi **(H)**; multifunctionality **(I)** and multidiversity **(J)** index of microorganisms. Uncontaminated original soil as control (CK). Low level (L, *RI* = 100) includes L1-L5 treatments. Moderate level (M, *RI* = 200) includes M1-M6 treatments. High level (H, *RI* = 400) includes H1-H6 treatments. ANOVA was used to determine whether the differences in various indexes are significant (*p* < 0.05) among different treatments at the same *RI* level.

At the same *RI* level, there were no significant changes among different treatments in the soil bacterial and fungal abundance, diversity indices and Multidiversity indices (Figure 1), which suggests that different proportions of Pb and Cd have minimal effect on microbial abundance and diversity. However, most studies showed that the toxicity of compound heavy metal pollution to microorganisms is greater than that of single element pollution (Song et al., 2018; Xu et al., 2019), and these studies have only focused on the effects of different concentrations of heavy metals on microorganisms and have ignored the role of TF of heavy metals. Therefore, the effect of compound pollution on microorganisms may be overestimated if the TF of heavy metals is not considered. It is essential to strongly consider the status of TF when assessing the risk of heavy metals to microorganisms.

Under different *RI* levels, there were significant differences in soil bacterial and fungal abundances, diversity indices and Multidiversity indices (*p* < 0.01; Supplementary Figure S2), which might have been caused by the stimulatory or inhibitory effects due to the different concentrations of heavy metal (Calabrese and Baldwin, 2003; Guo et al., 2020; Fan et al., 2021a). The concentrations of Pb and Cd were significantly correlated with the microbial parameters (Table 1), which were also found in other studies (Li et al., 2021; Shuaib et al., 2021). Compared to Pb and Cd, the *RI* demonstrated higher correlations with microbial parameters (Table 1), and the *RI* was more significantly correlated with fungal McIntosh, multidiversity and multifunctionality indices than with Pb and Cd (Supplementary Table S5). This result suggests that changes in microbial abundance and diversity are more dependent on *RI* levels than on different ratios of Pb and Cd. We also found that the *RI* level was negatively correlated with bacterial abundances and positively correlated with fungal ones (*p* < 0.01), which might be due to the different toxicological thresholds for bacteria and fungi (Singh et al., 2019; Fan et al., 2021b). In addition, the heavy metal stress changed the competition for resources among bacteria and fungi (Wang et al., 2010).

**Table 1:** The correlation of *RI*, Pb and Cd content with microbial abundance and diversity.

	**Bacteria**				**Fungi**				**Multidiversity**	
	**16S rRNA**	**Simpson**	**AWCD**	**McIntosh**	**ITS**	**Simpson**	**AWCD**	**McIntosh**	**Multidiversity**	**Multifunctionality**
RI	−0.791^**^	0.748^**^	−0.769^**^	−0.414^**^	0.758^**^	0.739^**^	−0.337^*^	−0.869^**^	0.876^**^	−0.840^**^
Pb	−0.418^**^	0.235	−0.428^**^	−0.054	0.598^**^	0.514^**^	−0.460^**^	−0.500^**^	0.454^**^	−0.385^**^
Cd	−0.421^**^	0.561^**^	−0.544^**^	−0.459^**^	0.319^*^	0.314^*^	−0.182	−0.484^**^	0.500^**^	−0.578^**^

** *Correlation is significant at the 0.01 level (2-tailed)*.

* *Correlation is significant at the 0.05 level (2-tailed)*.

In our research, we discovered that the TF of Pb and Cd were more appropriate for assessing the influence of heavy metals on microbial diversity and abundance than the concentrations of Pb and Cd. This study further elucidates the applicability of TF in soil microbial community risk assessment, especially in fungi. To improve the accuracy of heavy metal risk assessment, we should investigate the applicability of other heavy metals TF for microbes.

## Author Contributions

DL conducted experiment and wrote the main manuscript. JC helped with the experiment design and prepared data analytical methods. HZ helped with the writing and revision of the language. XZ helped with the soil microcosm experiment. JL was responsible for project administration and funding acquisition. All authors reviewed the manuscript.

## Conflict of Interest

The authors declare that the research was conducted in the absence of any commercial or financial relationships that could be construed as a potential conflict of interest.

## Publisher's Note

All claims expressed in this article are solely those of the authors and do not necessarily represent those of their affiliated organizations, or those of the publisher, the editors and the reviewers. Any product that may be evaluated in this article, or claim that may be made by its manufacturer, is not guaranteed or endorsed by the publisher.
