# Supplementary material for: Toxic Factors of Lead and Cadmium Fit in the Ecological Risk Assessment for Microorganisms
Source: Front Microbiol. 2022 Jun 24;13:927947. doi: 10.3389/fmicb.2022.927947 (PMC9263714; doi:10.3389/fmicb.2022.927947)
Supplement: Supplementary file 1 [file Table_1.DOCX]

**Table S1** **Basic properties of the soil used for the soil microcosm incubation**

| Soil physicochemical characteristics | Values |
| --- | --- |
| As (mg kg^-1^) | 26.01 ± 3.05 |
| Cd (mg kg^-1^) | 0.07 ± 0.02 |
| Cr (mg kg^-1^) | 102.89 ± 3.63 |
| Cu (mg kg^-1^) | 42.16 ± 4.93 |
| Ni (mg kg^-1^) | 36.89 ± 1.74 |
| Pb (mg kg^-1^) | 28.91 ± 0.78 |
| Zn (mg kg^-1^) | 97.84 ± 15.08 |
| Total nitrogen (TN) (g kg^-1^) | 79.63 ± 22.43 |
| Total carbon (TC) (g kg^-1^) | 14.27 ± 0.05 |
| Total sulfur (g kg^-1^) | 0.31 ± 0.08 |

Data are means ± standard deviation

**Table S2** **Classification of the potential ecological risk based on *RI* thresholds**

| *RI* thresholds | Degree of risk |
| --- | --- |
| *RI* < 150 | Low risk |
| 150 ≤ *RI* < 300 | Moderate risk |
| 300 ≤ *RI* <600 | Considerate risk |
| 600 ≤ *RI* | Very high risk |

**Table S3** **Treatments in the soil microcosm**

| *RI* levels | Simple | $E_{r}^{i}$ | The content of Pb/Cd/ (mg/kg) |
| --- | --- | --- | --- |
| CK | CK | Pb (5)/Cd (30) | Pb (28.81)/Cd (0.07) |
| L: *RI* = 100 | L1 | Pb (5)/Cd (95) | Pb (28.81)/Cd (0.21) |
|  | L2 | Pb (20)/Cd (80) | Pb (115.24)/Cd (0.19) |
|  | L3 | Pb (40)/Cd (60) | Pb (230.48)/Cd (0.14) |
|  | L4 | Pb (60)/Cd (40) | Pb (345.72)/Cd (0.09)/ |
|  | L5 | Pb (70)/Cd (30) | Pb (403.34)/Cd (0.07) |
| M: *RI* = 200 | M1 | Pb (5)/Cd (195) | Pb (28.81)/Cd (0.44) |
|  | M2 | Pb (40)/Cd (160) | Pb (230.48)/Cd (0.37) |
|  | M3 | Pb (80)/Cd (120) | Pb (460.96)/Cd (0.28) |
|  | M4 | Pb (120)/Cd (80) | Pb (691.44)/Cd (0.19) |
|  | M5 | Pb (160)/Cd (40) | Pb (921.92)/Cd (0.09) |
|  | M6 | Pb (170)/Cd (30) | Pb (979.54)/Cd (0.07) |
| H: *RI* = 400 | H1 | Pb (5)/Cd (395) | Pb (28.81)/Cd (0.88) |
|  | H2 | Pb (80)/Cd (320) | Pb (460.96)/Cd (0.75) |
|  | H3 | Pb (160)/Cd (240) | Pb (921.92)/Cd (0.56) |
|  | H4 | Pb (240)/Cd (160) | Pb (1382.88)/Cd (0.37) |
|  | H5 | Pb (320)/Cd (80) | Pb (1843.84)/Cd (0.19) |
|  | H6 | Pb (370)/Cd (30) | Pb (2131.94)/Cd (0.07) |

**Table S4** **Formulas for calculations of the diversity of soil microorganisms**

| Index | Formulae | Definitions |
| --- | --- | --- |
| Average well color development (AWCD) | $\mathrm{AWCD}=\frac{\sum_{i=1}^{N} {OD}_{i}}{N}$ | OD = C*_i_*-R, where C*_i_* is the absorbance value in each carbon source well and R is the absorbance value in the control well. while N is the number of substrates, which in this case is N = 31 (for Biolog ECO plate) or N = 95 (for Biolog FF plate) |
| Mclntosh diversity index (*U*) | $U=\sqrt{\sum_{i=1}^{N} {({OD}_{i})}^{2}}$ |  |
| Simpson diversity index (*D*) | $D=\frac{\sum_{i=1}^{S_{obs}} n_{i}(n_{i}-1)}{N\left( N-1 \right)}$ | S_obs_ = the total number of observed OTUs; n_i_ = the number of sequences in the OTU*i*; N = the total number of sequences |

**Table S5** **Comparison of the variability between correlation coefficients**

|  | Bacteria | | | | Fungi | | | | Multidiversity | |
| --- | --- | --- | --- | --- | --- | --- | --- | --- | --- | --- |
|  | 16S rRNA | Simpson | AWCD | McIntosh | ITS | Simpson | AWCD | McIntosh | multidiversity | multifunctionality |
| *RI*-Pb | 1.722 | **1.996*** | 1.535 | 1.058 | 0.826 | 1.041 | 0.401 | **2.135*** | **2.528*** | **2.221*** |
| *RI*-Cd | 1.712 | 0.915 | 1.118 | 0.152 | 1.810 | 1.707 | 0.456 | **2.193*** | **2.155*** | 1.520 |
| Pb-Cd | 0.010 | 1.081 | 0.417 | 1.210 | 0.985 | 0.666 | 0.858 | 0.058 | 0.373 | 0.701 |

**The way to do this is by transforming the correlation coefficient values into z scores. This transformation, also known as Fisher’s r to z transformation, is done so that the z scores can be compared and analyzed for statistical significance by determining the observed z test statistic. *** **Z score is significant at the 0.05 level (z score > 1.960)**

**
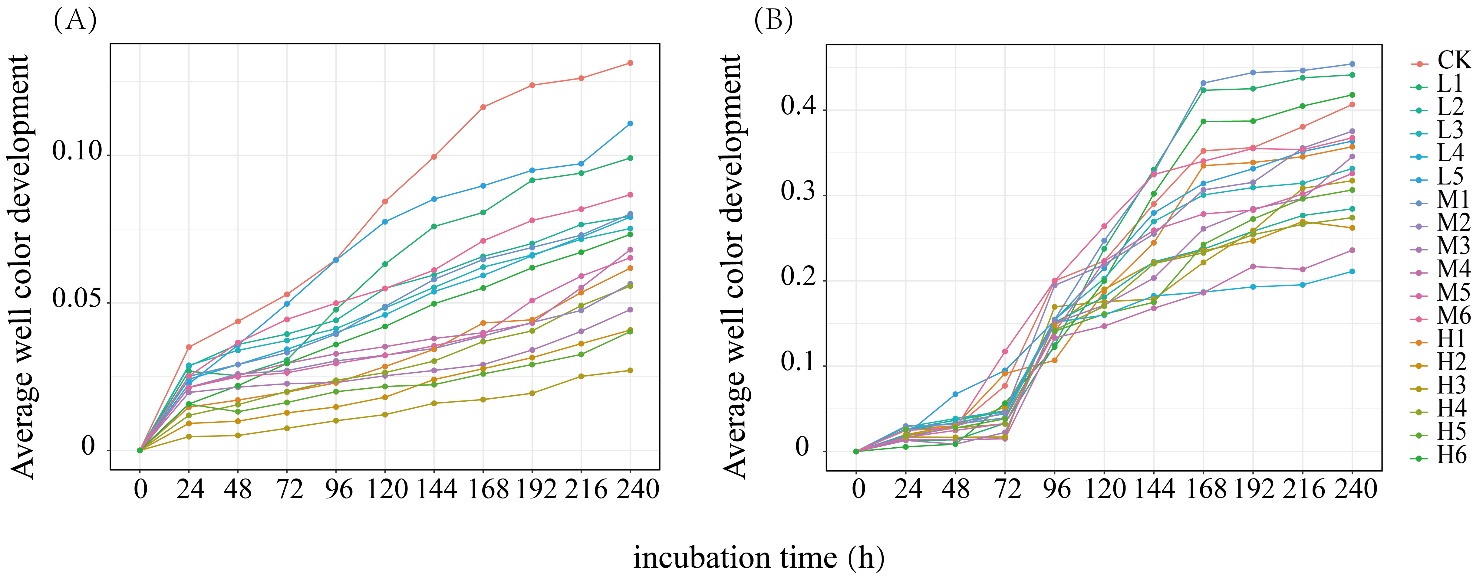
**

**Fig.S1** **Average well color development (AWCD) of metabolized substrates in** **Biolog Eco plate (A) and Biolog FF plate (B) based on 240 hours incubation. Low level (L, *RI* = 100) includes L1-L5 treatment. Moderate level (M, *RI* = 200) includes M1-M6 treatment. High level (H, *RI* = 400) includes H1-H6 treatment.**

**
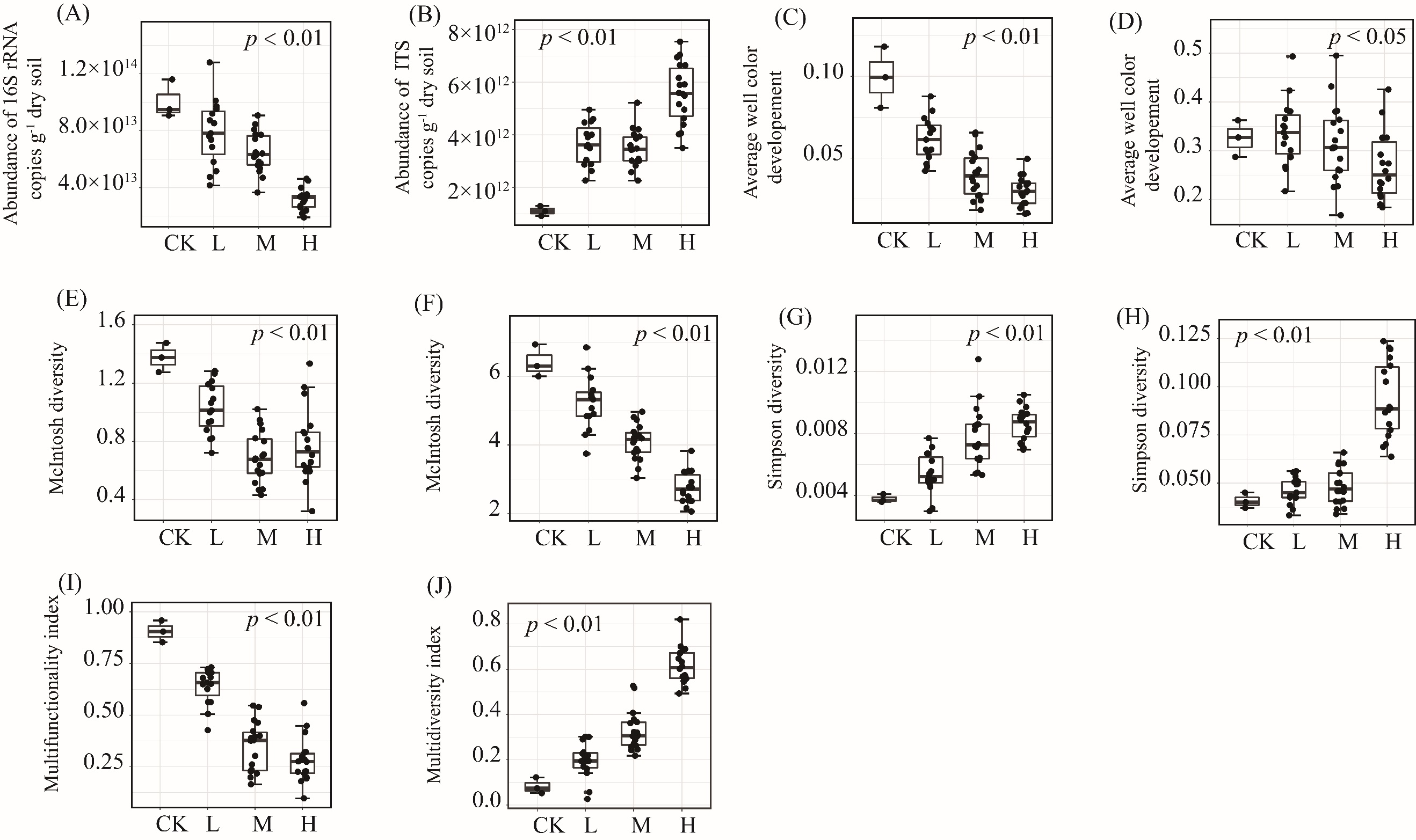
**

**Fig.S2** **Differences of microbial parameters under different *RI* levels. 16S rRNA (A) and ITS genes (B) abundance; Average well color development in** **Biolog Ecoplate (C) and FFplate (D); McIntosh diversity of bacteria (E) and fungi (F); Simpson diversity of bacteria (G) and fungi (H); multifunctionality (I) and multidiversity (J) indices of microorganisms.** **Uncontaminated original soil as control (CK). Low level (L, *RI* = 100) includes L1-L5 treatment. Moderate level (M, *RI* = 200) includes M1-M6 treatment. High level (H, *RI* = 400) includes H1-H6 treatment. ANOVA was used to determine whether the differences in various indices are significant (*p* < 0.05) among different treatments at the same *RI* level.**
